# Lipid Metabolic Pathways Confer the Immunosuppressive Function of Myeloid-Derived Suppressor Cells in Tumor

**DOI:** 10.3389/fimmu.2019.01399

**Published:** 2019-06-19

**Authors:** Dehong Yan, Adeleye O. Adeshakin, Meichen Xu, Lukman O. Afolabi, Guizhong Zhang, Youhai H. Chen, Xiaochun Wan

**Affiliations:** ^1^Shenzhen Laboratory for Human Antibody Engineering, Center for Antibody Drug Development, Shenzhen Institute of Advanced Technology, Chinese Academy of Sciences, Shenzhen, China; ^2^University of Chinese Academy of Sciences, Beijing, China; ^3^School of Life Science and Technology, Jinan University, Guangzhou, China; ^4^Department of Pathology and Laboratory Medicine, Perelman School of Medicine, University of Pennsylvania, Philadelphia, PA, United States

**Keywords:** MDSCs, lipid metabolism, FAO-OXPHOS, immunosuppressive, cancer immunotherapy

## Abstract

Myeloid-derived suppressor cells (MDSCs) play crucial roles in tumorigenesis and their inhibition is critical for successful cancer immunotherapy. MDSCs undergo metabolic reprogramming from glycolysis to fatty acid oxidation (FAO) and oxidative phosphorylation led by lipid accumulation in tumor. Increased exogenous fatty acid uptake by tumor MDSCs enhance their immunosuppressive activity on T-cells thus promoting tumor progression. Tumor-infiltrating MDSCs in mice may prefer FAO over glycolysis as a primary source of energy while treatment with FAO inhibitors improved anti-tumor immunity. This review highlights the immunosuppressive functions of lipid metabolism and its signaling pathways on MDSCs in the tumor microenvironment. The manipulation of these pathways in MDSCs is relevant to understand the tumor microenvironment therefore, could provide novel therapeutic approaches to enhance cancer immunotherapy.

## Introduction

Myeloid-derived suppressor cells (MDSCs) are pathologically activated cells displaying an exceptional immunosuppressive ability (1, 2). They rapidly expand in cancer, trauma, infectious, autoimmune, and graft vs. host disease (3–8). MDSCs are phenotypically similar to monocytes and neutrophils, thus are further divided into two subsets; monocytic MDSCs (M-MDSCs) and polymorphonuclear MDSCs (PMN-MDSCs) respectively (9, 10). Generally, the cell surface markers for MDSCs include Gr1 and CD11b in mice (11, 12). The M-MDSCs are characterized by CD11b^+^Ly6C^high^Ly6G^−^ unlike PMN-MDSCs which are CD11b^+^Ly6C^low^Ly6G^+^ (10, 11). Human M-MDSCs on the other hand, are CD33^high^CD14^+^CD15^+^/CD66b^−^HLA-DR^−/low^, whereas PMN-MDSCs are characterized by CD33^dim^CD14^−^CD15^+^/CD66b^+^HLA-DR^−^ (13, 14). More importantly, these cells potently suppress innate and adaptive immunity. Thus, are considered a promising therapeutic target in cancer immunotherapy.

Metabolic reprogramming has been reported to be a crucial factor in the alteration of MDSCs function (15–20). Lipids which maintain cell membrane integrity, homeostasis, signaling, and healthy performance have been implicated to modulate the function of MDSCs (21–23). Recently, uncontrolled lipid accumulation was found to be higher in MDSCs from cancer patients and mice with an established tumor compared with tumor-free counterparts (24–26). This increased the immunosuppressive activity in the hyperlipidemic tumor-bearing mice and impaired T-cell activation (24, 25, 27). In this review, we discuss the roles of lipid in modulating MDSCs function and its related metabolic pathways. Further understanding of the biochemical pathways involved in lipid manipulation of MDSCs is pertinent to understand the tumor microenvironment and improve chemo- and immuno-therapies.

## An Overview of Lipid Metabolism In MDSCs

**Figure 1:**
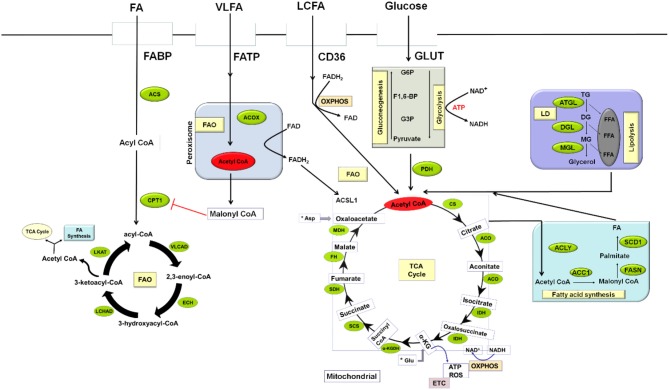
An overview of MDSCs Lipid Metabolism in a tumor environment. Lipid metabolism in MDSCs can undergo two processes: fatty-acid synthesis and fatty-acid β-oxidation. Fatty acid synthesis takes place in the cytosol while β-oxidation occurs in the mitochondrial. Several metabolic networks regulate the activation and survival of MDSCs to enhance tumor proliferation. Glycolysis, the breakdown of glucose to pyruvate with the concomitant release of ATP taking place in the cytosol is the major source of energy to most cells. In the mitochondrial, PDH converts pyruvate to acetyl CoA, the central dogma of metabolism which has several metabolic fates, including TCA cycle, oxidative phosphorylation, and fatty acid biosynthesis. ACC1, Acetyl CoA carboxylase; ACLY, ATP citrate lyase; ACO, Aconitase; ACOX, Acyl coA oxidase; ACS, Acyl CoA synthase; ACSL1, long-chain acyl-CoA synthetase isoform 1; Asp, Aspartate; ATGL, Adipose triglyceride lipase; ATP, Adenosine triphosphate; CD36, Cluster of differentiation 36; CPT, Carnitine palmitoyltransferase 1; DG, Diglyceride; DGL, Diglyceride lipase; ECH, 2, 3-enoyl-CoA hydratase; ETC –Electron transport chain; F1,6-BP, Fructose-1,6-bisphosphate; FA, Fatty acid; FABP, Fatty acid-binding protein; FAD, Flavin adenine dinucleotide; FADH2, Reduced FAD; OXPHOS, Oxidative phosphorylation; FAO, Fatty acid oxidation; FASN, Fatty acid synthase; FATP, Fatty acid transport protein; FFA, Free fatty acid; FH, Fumarate hydratase; G3P, Glyceraldehyde-3-phosphate; Glu, Glutamate; GLUT, Glucose transporter; GP6, Glucose-6-phosphate; IDH, Isocitrate dehydrogenase; LCHAD, Long-chain 3-hydroxyacyl-CoA dehydrogenase; LD, Lipid droplet; LKAT, long chain 3-ketoacyl-CoA thiolase; MDH, Malate dehydrogenase; MG, Monoglyceride; MGL, Monoglyceride lipase; NAD, Nicotinamide adenine dinucleotide; PDH, Pyruvate dehydrogenase; ROS, Reactive Oxygen species; SCD1, Stearoyl-CoA desaturase 1; SCS, Succinyl CoA synthase; SDH, Succinate dehydrogenase; TCA, Tricarboxylic acid; TG, Triglyceride; VLCAD, Very-long-chain acyl-CoA dehydrogenase; VLFA, Very long chain fatty acid; LCFA, Long chain fatty acid; α-KG, Alpha-ketoglutarate; α-KGDH, Alpha ketoglutarate dehydrogenase.

Acetyl CoA, a major intermediate in several biochemical processes plays a pivotal role in lipid metabolism. It is the primary building blocks for biosynthesis of fatty acid, cholesterol, and the end product of fatty acid oxidation (FAO). Lipid catabolism involves the oxidation of long-chain fatty acids, which takes place in the mitochondrial via the transportation of lipids from the cytosol by the carnitine palmitoyltransferase system. Carnitine palmitoyltransferase 1 (CPT1) catalyzes the rate-limiting step in FAO. During fatty acid oxidation, the continuous elimination of 2-carbon units from the β-position of fatty acyl-CoA molecule produces acetyl CoA which sustains oxidative phosphorylation (OXPHOS) and tricarboxylic acid cycle (TCA) in the cell (28, 29). In contrast to FAO, synthesis of fatty acid occurs in the cytosol; commencing with the carboxylation of acetyl CoA to malonyl CoA in an ATP-dependent manner catalyzed by acetyl CoA carboxylase 1 (ACC1), the reaction rate determining enzyme (Figure 1). This is followed by the condensation of another molecule of acetyl CoA with the malonyl CoA to produce saturated long chain fatty acids in a process catalyzed by fatty acid synthase (FASN). These steps lead to the formation of other complex lipids like phospholipids, cholesterol esters, and triglycerides.

## Energy Metabolic Pathways of MDSCs

It is important to note that nearly all major biomolecules (carbohydrates, proteins, and lipids) are converted to a common intermediate in the form of acetyl CoA. Acetyl CoA can be further oxidized to CO_2_ or take part in some other biosynthetic pathways as required by the cells. Production of adenosine triphosphate (ATP) to enhance cellular functions, survival, and synthesis of intermediates allowing for cellular growth and proliferation by immune cells rely on various energy metabolic pathways (30, 31). Interconnection of metabolic network (glycolysis, the TCA cycle, and OXPHOS) plays a crucial role in fulfilling these energy needs (Figure 1). Glycolysis occurs in the cytosol while the TCA cycle and OXPHOS are restricted to the mitochondria. Glycolysis commences with glucose uptake from the extracellular environment via GLUT; glucose is then phosphorylated to glucose-6- phosphate (G6P) by hexokinases. G6P is further processed to pyruvate via multiple enzyme-catalyzed reactions during which it reduces NAD^+^ to NADH to yield 2 molecules of ATP. Under normoxia, glycolysis-derived pyruvate is converted into acetyl-CoA in a reaction regulated by pyruvate dehydrogenase (PDH) complex. This acetyl CoA condenses with oxaloacetate to form citrate in a reaction catalyzed by citrate synthase in the TCA cycle. This cycle produces NADH and FADH2 and transfers electrons generated through the electron transport chain (ETC) to fuel OXPHOS to yield 30–36 molecules of ATP per molecule of glucose. Cells can also use fatty acid through FAO, which yields acetyl-CoA to sustain the TCA cycle and OXPHOS; this can facilitate the generation of substantial amounts of ATP (over a 100 ATP molecules per molecule of palmitate). Most importantly, cells to a varying extent can select their preferred metabolic pathways among several available intermediates to produce ATP. In the immune cells, nutrient, and oxygen availability which can be controlled by growth factors and cytokines, as well as important receptor signaling events, regulate the metabolic fate of these cells.

Certain key enzymes derived from MDSCs are important to their suppressive role, these enzymes deplete the essential amino acids required for T-cell function and proliferation (32). Increased arginase 1 (ARG1) expression in MDSCs depletes L-arginine needed for T-cell functions (33). Also, MDSCs accumulation deplete L-cysteine levels via its sequestration and consumption (34). The depletion of these amino acids results in downregulation of ζ-chain in the T cell receptor (TCR) thus inhibiting proliferation of antigen-specific T-cells. Similarly, MDSCs express the inducible enzyme Indoleamine 2, 3-dioxygenase (IDO), which catalyzes tryptophan metabolism through the kynurenine pathway (35, 36). Thus, IDO expression leads to tryptophan deprivation and induces regulatory T-cells (Tregs) expansion which represses T-cells (37, 38). While the pivotal role of nitrogen metabolism in mediating the immunosuppressive function of MDSCs on T-cells in tumors is well-established (32), little is known about other metabolic pathways in these cells. Carbon metabolism (glycolysis, pentose phosphate pathway (PPP), TCA, FAO pathways) and its crosstalk with nitrogen metabolism during MDSCs maturation in tumors need to be expounded.

FAO and glycolysis are crucial pathways in tumor growth (39), however, it is not known whether MDSCs prefer FAO over glycolysis. It was previously reported that tumor-infiltrating MDSCs (M-MDSCs and PMN-MDSCs) in comparison with peripheral MDSCs and murine myeloid cells prefer FAO as their energy sources (27). This was deduced from the observed elevated mitochondrial mass, increased oxygen consumption rate (OCR) and upregulation of crucial FAO regulatory enzymes [acyl-CoA dehydrogenase (ACADM), CPT1, 3-hydroxyacyl-CoA dehydrogenase (HADHA), and peroxisome proliferator-activated receptor gamma coactivator 1-beta (PGC1β)] in PMN-MDSCs. The study revealed a correlation between expression of FAO genes (such as CPT1 and HADHA) and fatty acid uptake in patient-derived tumor-infiltrating MDSCs (22, 27). In the study, both extracellular acidification rate (ECAR) and OCR were elevated, indicating an overall metabolic alteration. Although the ratio of OCR/ECAR was increased, suggesting that FAO may be preferred to glycolysis in tumor-infiltrating MDSCs from Lewis lung carcinoma.

In addition, Jian et al., recently reported that ECAR and glycolytic enzymes are upregulated in total MDSCs. Whereas, PMN-MDSCs were observed to utilize both glycolysis and oxidative phosphorylation to produce energy for its suppressive role due to the elevated metabolic state of the tumor-bearing host (40). Inhibition of two key enzymes in glycolysis: hexokinase (HK) and glyceraldehyde-3-phosphate dehydrogenase (GAPDH) by 2-deoxyglucose (2-DG) and sodium iodoacetate (IA), respectively, reduced MDSCs expansion, leading to a delay in tumor progression via induction of ROS-mediated apoptosis of MDSCs (40).

Another study reported that latent membrane protein 1 (LMP1) associated with Epstein-Barr virus mediates glycolysis by upregulating GLUT1 in tumor (41). This promotes the induction of GM-CSF, IL-6, and IL-1β production through COX-2 and NLRP3 inflammasome signaling pathways to enhance MDSCs differentiation and expansion, thereby promoting nasopharyngeal carcinoma (NPC) progression (41). However, a more recent study reported that EBV-encoded LMP1 induces *de novo* lipogenesis and lipid droplets formation through the activation of sterol regulatory element-binding protein 1 (SREBP1) which promotes progression of NPC (42). This suggests that LMP1 could also mediate other metabolic pathways such as lipogenesis (previously reported) or FAO to regulate MDSCs alteration in NPC progression. Therefore, a comprehensive study on the role of LMP1 expression in regulating immune cells (especially MDSC) in tumor state could help broaden understanding of the most upregulated pathway in MDSCs.

A recent study reported the correlation between MDSCs and glycolysis in human triple negative breast cancer (TNBC) and observed that restriction of glucose metabolism inhibits G-CSF and GM-CSF expression (43). This resulted in reduced MDSCs number while conferring tumor immunity by enhancing T-cell function. MDSCs are able to utilize anaerobic glycolysis when oxygen supply is limited to enhance their immunosuppressive role in the tumor microenvironment (44). This was observed by the upregulation of lactate dehydrogenase A (LDHA) (43), an enzyme involved in the reversible reaction of pyruvate to lactic acid. This could be an indicator of highly proliferative and energy demanding cell for the production of NAD^+^ in subsequent ATP generation when oxidative phosphorylation is restricted due to insufficient oxygen availability. Inhibition of LDHA in a murine pancreatic cancer model decreased MDSCs frequency in the spleen and enhanced cytolytic activity of natural killer (NK) cells (44). Extrinsic lactic acid also increased the proportion of MDSCs derived from bone marrow (BM) cultured cells in the presence of GM-CSF and IL-6. Furthermore, MDSCs undergoing anaerobic glycolysis partly oxidize L-glutamine to provide a favorable condition for tumor growth (45). Although anaerobic glycolysis occurs 100 times faster than oxidative phosphorylation, it is less efficient and only helps in fulfilling a short-term energy requirement when oxygen supply is low (46).

Based on the diversity and dynamic attributes of the tumor milieu across various cancers as well as the stage of progression of same cancer, it is possible that the process of nutrient metabolism in immune cells might also differ across these conditions (39, 47). Recent studies have reported that the switch between glycolysis and oxidative phosphorylation in tumor-associated macrophages (TAM) is dependent on the stages of cancer development (48, 49). In relation to TAM, MDSCs also exhibit a certain degree of plasticity and may adopt a typically activated (M1) or alternatively activated (M2) phenotype, with antitumor or tumor-promoting roles, respectively (50). Therefore, the alterations of MDSCs differentiation, maturation and function may rely on overall central carbon metabolism and upregulation of cellular bioenergetics fluxes (45).

So far, the metabolic preference of MDSCs in tumor microenvironments is not fully known and requires more robust investigations. However, current evidence suggests that it may involve global regulation of metabolic flux.

## Oxidized Lipids Regulate MDSCs Function in the Tumor Microenvironment

**Figure 2:**
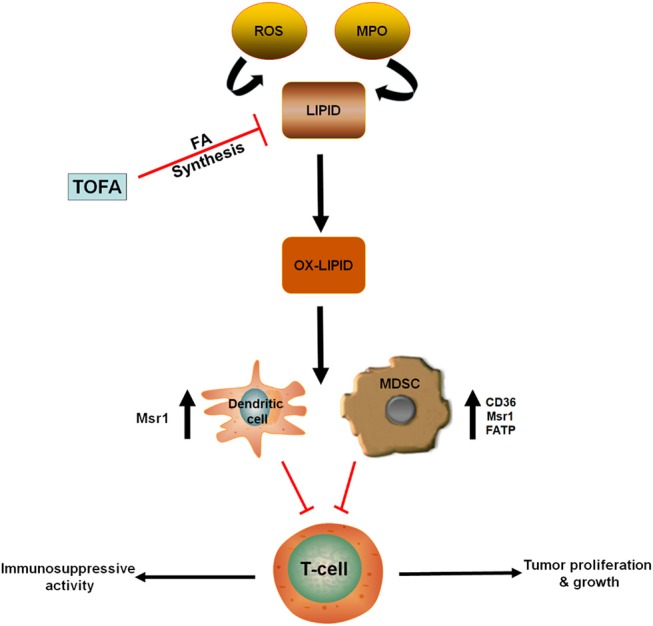
Oxidized lipids contribute to the immunosuppressive role of MDSCs and DC. ROS and MPO contribute to the oxidation of lipid accumulated in antigen presenting cells (DC) and MDSCs. In these cells, upregulation of lipid transporters (CD36, Msr1, FATP) increase fatty acid uptake. Hence, promoting immunosuppressive activity and reducing T-cell function. However, treatment with TOFA (fatty acid synthesis inhibitor) blocked the accumulation of lipid in both DC and MDSCs. CD36, Cluster of differentiation 36; DC, Dendritic cell; FATP, Fatty acid transport protein; MDSCs, Myeloid-derived suppressor cells; MPO, Myeloperoxidase; Msr1, Macrophage scavenger receptor 1; Ox-lipid, Oxidized lipid; ROS, Reactive oxygen species; TOFA - 5, (tetradecycloxy)-2-furoic acid.

Utilization of oxidized lipid as an energy source is crucial to the immunosuppressive roles of MDSCs in the tumor microenvironment (24). Gabrilovich et al. demonstrated that accumulation of oxidized lipids in tumor-infiltrating CD11c^+^ DCs blocks antigen presentation and their orientation on major histocompatibility complex (MHC) class II (51, 52). This, in turn, blocks antigen-mediated cross-presentation and inhibits T cell stimulation. They also showed that targeting ACC1 with 5- (tetradecycloxy)-2-furoic acid (TOFA), reverses the effects of lipids, suggesting that the fatty acid biosynthesis pathway is involved in this process (Figure 2) (51).

In line with other myeloid cells, substantial lipid accumulation was observed in tumor-derived MDSCs (24, 53). MDSCs with lipid overload demonstrated greater immunosuppressive effect on CD8^+^ T cells, compared to MDSCs with normal lipid content. Lipid accumulation in tumor-derived MDSCs can be linked to an increase in fatty acid uptake. This is supported by the study of Cao et al., which revealed an increased expression of fatty acid transport protein 4 (FATP4) in murine tumor-derived MDSCs (53). Most of the lipids detected in the MDSCs of tumor-bearing mice and cancer patients were found to be oxidized (Figure 2), possibly resulting from the oxidative activities of reactive oxygen species (ROS) and myeloperoxidase (MPO) (24, 54). Inhibition of ROS and MPO in these cells almost completely expunged the oxidation of lipids and resulted in MDSCs with a diminished immunosuppressive activity (24).

A recent study identified the upregulation of FATP2 on PMN-MDSC as a critical regulator of their immunosuppressive function (26). FATP2 promotes the accumulation of arachidonic acid leading to prostaglandin E2 (PGE2) synthesis in PMN-MDSCs thereby boosting their immunosuppressive activities. Thus, the pharmacological inhibition of FATP2 could serve as a novel and targeted therapeutic strategy to block the immunosuppressive activity of PMN-MDSCs. Collectively, these studies suggest the critical role of oxidized lipids in regulating myeloid cells function and specifically in MDSC as a potential therapeutic target in cancer.

## Exogenous Fatty Acid Uptake Enhances Suppressive Activity In MDSCs

MDSCs take up fatty acids from the tumor microenvironment and utilize them via several pathways. Our group previously reported that polyunsaturated fatty acids (PUFAs) impaired myeloid cell differentiation in bone marrow from tumor-bearing mice thus promoting the accumulation and functional activity of MDSCs (55). The study further demonstrated that dietary intake of linoleic acid (LA) and alpha-linolenic acid (ALA) promoted tumor growth in agreement with the observation by another group (56). It was recently discovered that culturing MSC-2, a myeloid suppressor cell line in the presence of long chain unsaturated fatty acids, oleate and linoleate increased lipid droplet accumulation which in-turn suppressed T-cell activity (57). However, T-cell activation remained unaffected in MSC-2 cells cultured in the presence of stearate, a saturated fatty acid that also accumulated lipid droplets. Removal of oleate from the culture medium triggered the mobilization of lipid droplets in this cell-line, thereby diminishing its immunosuppressive activity. Furthermore, it was observed that inhibiting diacylglycerol acyltransferases (DGAT) abolishes oleate-induced lipid droplet formation and impaired the immunosuppressive activity of MSC-2 (57). In addition, another group of researchers reported that MDSCs treated with linoleic acid demonstrated a stronger inhibitory effect on T-cell compared to those treated with palmitic acid which is a saturated fatty acid (53). In summary, unsaturated fatty acids which are known to be more susceptible to oxidation, contribute to the suppressive ability of MDSCs during cancer via upregulation of lipid metabolic gene such as DGAT.

## Signaling Pathways Involved in Lipid Metabolism of MDSCs

Recent findings suggest a relationship between oxidative phosphorylation initiated by lipid metabolism and its contribution to immunosuppressive myeloid cells. However, the underlying molecular mechanisms associated with FAO in subpopulations of tumor-infiltrating MDSCs is yet to be fully elucidated (54, 58). The role of kinases like AMPK and PI3K, transcription factors such as STATs, enzymes involved in FAO as well as several receptors including Peroxisome proliferator-activator receptors (PPARs), among others on immune cells, including MDSC, has been described in different *in vitro* and *in vivo* models. For example, pharmacological inhibition of STAT3 and STAT5 (activated by tumor-derived cytokines) decreased lipid accumulation, mitochondrial metabolism, and immunosuppressive function in MDSCs in an *in vitro* study (25). Increased expression of crucial genes encoding enzymes in FAO is linked to the suppressive role of tumor MDSCs which was abolished by FAO inhibitors (27). More detailed description of those critical components of the signaling pathways involved in the cellular lipid metabolism is described below.

### LXR

**Figure 3:**
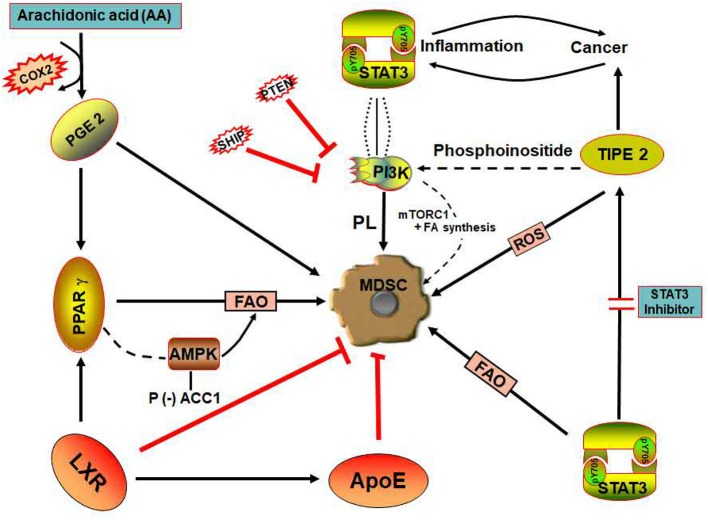
Signaling pathways involved in lipid metabolism of MDSCs. (i) SHIP and PTEN are negative regulators of PI3K/AKT—involved in the promotion of lipid and sterol synthesis (ii) COX-2 is the enzyme which catalyzes arachidonic acid into PGE2, a pro-inflammatory lipid mediator that could result in elevated MDSCs. (iii) PPAR-γ initiates AMPK activation, thereby promoting FAO in MDSCs to enhance its immunosuppressive ability. (iv) LXR is a nuclear hormone receptor that regulates lipid homeostasis and enhances the transcriptional activation of ApoE—involved in lipoprotein metabolism. LXR inhibits MDSCs suppressive activity on T-cells. (v) STAT3 signaling enhances FAO and also upregulates TIPE2 expression in MDSCs. (vi) TIPE2, a promoter of the immunosuppressive function of MDSCs, regulates PI3K via signaling of phosphoinositide and can be inhibited by STAT3 inhibitors. ACC1, Acetyl CoA carboxylase 1; AMPK, AMP-activated protein kinase; APOE, Apolipoprotein E; COX-2, Cycloxygenase 2; FAO, Fatty acid oxidation; LXR, Liver X receptors; MDSCs, Myeloid-derived suppressor cells; mTORC1, mammalian target of rapamycin complex 1; PGE2, Prostaglandin E2; PI3K, Phosphoinositide-3-Kinase; PL, Phospholipid; PPARγ, Peroxisome proliferator-activator receptors gamma; PTEN, Phosphatase and tensin; ROS, Reactive oxygen species; SHIP−5-inositol phosphatase; STAT 3, Signal transducer and activator of transcription; TIPE 2, Tumor necrosis factor alpha-induced protein 8 like 2; P, Phosphorylation; +, Stimulate; –, Deactivation.

Liver X receptors comprise two isoforms, LXRα and LXRβ which are encoded by *Nr1h3* and *Nr1h2*, respectively. Both isoforms are members of the nuclear hormone receptor family that modulate several transcriptional factors. LXR acts as a critical regulator of lipid homeostasis (Figure 3) by driving the expression of key genes involved in cholesterol, fatty acid, and glucose metabolism (59, 60).

Masoud et al., reported the effect of LXR activation on MDSCs expansion and their immunosuppressive activities on T-cell stimulation both *in vivo* and *in vitro* (61). RGX-104, an LXR agonist, significantly decreased the abundance of PMN-MDSCs and M-MDSCs from B16F10 melanoma tumor-bearing mice. The proportion of PMN-MDSCs generated *in vitro* from bone marrow cells treated with LXR agonist was decreased in the presence of GM-CSF. Oral administration of RGX-104 also decreased the population of MDSCs in cancer patients. It was previously reported that cholesterol-induced LXR sumoylation blocks IL-9 expression in CD8^+^ T cells, partially by reducing the binding of NF-κB p65 subunit to IL-9 promoter (62). IL-9 demonstrates a critical role in the antitumor response of CD8^+^ T cell subset (Tc9) (62–64). Also, IL-9 expressing T-cells have been identified in humans (65) and it was reported to enhance the function and survival of human tumor-infiltrating T-cells (66). It is imperative to decipher how the accumulation of lipids or exogenous fatty acid uptake regulates LXR signaling pathways in immune cells.

LXR promotes the transcriptional activation of a secretory protein, apolipoprotein E (ApoE) (61), which mediates the cellular uptake of lipoprotein particles by binding to low-density lipoprotein receptor (LDLR) and chylomicron remnants receptor (67). ApoE interaction with these receptors activates lipid (such as cholesterol, phospholipid, and triglycerides) metabolic pathways (68). A recent study showed that ApoE regulates MDSCs survival and tumor progression; ApoE^−/−^ mice had increased levels of PMN-MDSCs and M-MDSCs. MDSCs from ApoE deficient mice showed reduced T-cell proliferation *in vitro* (61). Since ApoE plays a crucial role in lipoprotein metabolism (69), its deletion could enhance MDSCs immunosuppressive activity on T-cells via lipid accumulation. Al-Khami et al., reported the accumulation of lipid in bone-marrow derived MDSCs following extracellular uptake of low-density lipoproteins (LDL) and very low-density lipoproteins (VLDL) (25). This was observed to enhance oxidative metabolism and upregulation of ARG1 in MDSCs. Altogether, these suggest that the regulation of lipid metabolism via ApoE expression may alter MDSCs function in the tumor milieu.

### PPARs

Peroxisome proliferator-activator receptors (PPARs) are “lipid sensing” nuclear receptors activated by free fatty acid (FFA), prostaglandins, eicosanoids, or sterols (70). They are divided into three subtypes which are: PPARα, PPARγ, and PPARδ. PPARγ and PPARδ elicit the expression of certain FAO genes (Figure 3) and coordinate anti-inflammatory functions while PPARα directly upregulates the expression of CPT1a (71), a crucial enzyme involved in mitochondrial fatty acid oxidation.

PPARγ plays an important role in regulating lysosomal acid lipase (LAL) activity, a key enzyme in the metabolism of neutral lipids. LAL^−/−^ MDSCs demonstrate greater immunosuppression thereby promoting tumor cell proliferation, growth and metastasis. Activation of PPARγ pathway in LAL^−/−^ MDSCs impaired tumor growth and metastasis *in vivo* as well as *in vitro* (72). Cardiolipin, a phospholipid, promotes IL-10 expression in MDSCs from the lungs of tumor-bearing mice by activation of PPARγ activity (70, 73) but this can be inhibited by GW9662 (a specific inhibitor of PPARγ) (74). However, the roles of the PPAR family in tumor biology remain unclear.

PPARα was reported to mediate the transcription initiation of CPT gene in CD4^+^ T-cells isolated from Jurkat cell and a murine model of non-alcoholic fatty liver disease (NAFLD, a risk factor of hepatocellular carcinoma) (75) treated with linoleic acid. PPARα agonist, benzofibrate, increased the generation of mitochondrial ROS and induced apoptosis by upregulating CPT1a following treatment with LA in murine and Jurkat cell. This effect was reversed in the presence of PPARα inhibitor (GW6471), illuminating the important role PPARα may play in regulating CPT1 (76).

Furthermore, PPARα agonist, fenofibrate, enhanced fatty acid catabolism in CD8^+^ T-cell under hypoxia and low glucose condition (77) thereby activating genes encoding proteins (such as PPARα and CPT1a) involved in lipid metabolism and TCA cycle. Vaccinated animals to elicit melanoma-specific CD8^+^ TILs response and treated with fenofibrate significantly delayed tumor progression, confirming that enhanced fatty acid catabolism improves CD8^+^ TILs functions. In PPARα KO mice, CD8^+^ cells cultured in glucose-deprived media exhibited a reduction in the transcript for fatty acid metabolism and lower functionality in comparison with the wild-type cells (77). This study suggests fatty acid catabolism is essential for CD8^+^ TILs functions when access to glucose is limited. Therefore, targeting PPARs pathways could be another promising option in manipulating lipid content in MDSCs for successful cancer therapy.

### AMPK

AMP-activated protein kinase (AMPK) is a potential mediator of lipid metabolism regulating cellular homeostasis in MDSCs (45). Activation of PGC1β/PPARγ axis induces AMPK signaling, demonstrating a crucial role in mitochondrial biogenesis; thereby activating the expression of genes encoding proteins involved in FAO (15). More so, AMPK regulates the phosphorylation and deactivation of ACC1, the key enzyme involved in the regulation of fatty acid synthesis (78) (Figure 3).

MDSC-mediated AMPK phosphorylation could increase survival of multiple myeloma cells. Treatment with compound C (AMPK inhibitor) in the presence or absence of MDSCs decreased AMPK phosphorylation and induced apoptosis of multiple myeloma cells (79). Oxidative stress and upregulation of HIF-1α were reported as triggers of AMPK activation in osteosarcoma cells (80, 81). Since MDSCs from tumor-bearing mice and cancer patients demonstrate a high amount of intracellular ROS (40, 82–84), it is possible that accumulated ROS may enhance AMPK activity. In addition, cytokines such as IL-10 and TGF-β are elevated in MDSCs (85, 86) and may also induce AMPK phosphorylation.

Although the role of AMPK activation in cancer is widely studied and considered as a tumor suppressor [reviewed in (87)], its activity in modulating MDSCs function remains unclear. There have been conflicting reports on the exact role of AMPK in modulating MDSCs activity. It was recently demonstrated that pharmacological targeting of AMPK abrogates MDSCs function in tumors by repressing the expression of iNOS, arginase, and promoting T-cell proliferation (88, 89). Another report suggested that AMPK signaling enhances MDSCs immunosuppressive activity in doxorubicin-resistant tumors via upregulation of miR-10a expression (90). However, inhibition of miR-10a abrogated the elevated CD11b^+^Gr1^+^ MDSCs frequency and also M2 signature genes such as ARG1, TGF-β, and MMP9. Hence, there is a need for more comprehensive studies to elucidate the role of this potential lipid metabolic mediator on MDSCs function in the tumor microenvironment.

### PI3K/AKT/mTOR

Phosphoinositide-3-Kinase (PI3K) signaling performs an important role in regulating cellular functions and coordinate processes such as protein synthesis, glucose homeostasis, cellular metabolism, cell growth, migration, and survival (91). PI3K catalyzes the phosphorylation of phosphatidylinositol in the plasma membrane by adding a phosphate moiety to the 3'OH on the lipid (92). Several scientists investigating the etiology of cancer at the molecular level have intensely studied this pathway due to its frequent alteration in cancer (93). PI3K has been reported to regulate physiological activities in neutrophils (94, 95). In aging mice, bone marrow and secondary lymphoid organs accumulate a substantial level of MDSCs, which may be associated with altered PI3K/AKT signaling pathway. The molecular mechanism enhancing the suppressive activity of MDSCs revealed an upregulation in iNOS activity and inhibition of T-cell activation. This leads to an alteration in the immune system and supports immune senescence (96). These observations made PI3K signaling pathway a novel target for new cancer therapy (97) and we consider its signaling may be a potential modulator of lipid accumulation in MDSCs to enhance its suppressive activity on T-cells proliferation.

Phosphatase and tensin (PTEN) and 5-inositol phosphatase (SHIP) are negative regulators of the PI3K/AKT signaling pathway (Figure 3) regulating phosphoinositide metabolism in immune cells (98). SHIP suppresses cell growth and survival via the movement of cell membranes (shortly after stimulation of the extracellular compartment) leading to the conversion of phosphatidylinositol-3,4,5-triphosphate (PIP3) into phosphatidylinositol-3, 4- bisphosphate (PI3,4-P2), thus inhibiting PI3K (99). The MDSCs population can be increased through the downregulation of SHIP expression by cancer cells secreting factors such as GM-CSF, IL-6, and TGF-β. It was previously reported that increased MDSCs population in SHIP^−/−^ tumor-bearing mice lymphoid compartment contributes to immunosuppressive allogenic T-cell responses *in vitro* and *in vivo* (100). Hence, treatment strategies toward enhancing the activity of SHIP could mitigate the immunosuppressive effect of MDSCs and serve as therapeutic approaches in cancer (101).

The mammalian target of rapamycin (mTOR) is a major component of the PI3K/AKT pathway involved in cell proliferation and nutrient availability. It also controls the innate and adaptive immune response in multiple immune cells (102). Inhibition of mTOR signaling with rapamycin-induced the upregulation of arginase-1 and iNOS in MDSCs (103). Thus, enhancing MDSCs immunosuppressive activity and reduced T-cells proliferation. Constitutive initiation of Akt has been documented to promote lipid and sterol synthesis in addition to glycolysis (104, 105). Given that genetic manipulation of mTORC1 (either by deletion of TSC1 or TSC2 in fibroblast) activate downstream transcription factors involved in lipid biosynthesis (106), the signaling pathway via Akt-TSC-mTORC1-S6K1-SREBP axis could be a possible pathway in MDSC. When mTORC1 is constitutively active, the transcription factor, SREBP is activated and drives the expression of sterol and fatty acid biosynthesis genes (106). The role of mTORC2 in lipid regulation unlike mTORC1 still remains unclear. Chen et al., recently highlighted another mTORC2 target, ATP citrate lyase (ACLY) in lipid metabolism. It converts citrate derived from the TCA cycle into acetyl CoA in the cytoplasm where it can be used for lipid biosynthesis. ACLY was identified as a target of mTORC2 in a breast cancer cell line; revealing that mTORC2 and not mTORC1 is necessary for the generation of acetyl CoA in an ACLY-catalyzed reaction (107). Inhibition of ACLY or mTORC2 activity altered mitochondrial function by reducing cell proliferation and tumor growth (108). Since mTORC2 can regulate lipid metabolism by limiting the activity of ACLY to generate the building blocks of lipids, targeting this signaling pathway could attenuate the immunosuppressive activities of MDSCs.

Increased mRNA and protein synthesis resulting from the phosphorylation of S6K and 4EBP1 proteins via mTOR enhanced cell proliferation (109). Reports had shown the possible regulatory role of the mTOR pathway during inflammatory responses by an alteration in the activity of STAT3 and NF-κB in myeloid cells (110). Chen et al., showed the engagement of the mTOR signaling pathway in monocytes differentiation to TAM (111). The effect of mTOR signaling in promoting the expression of lipid and sterol genes when they are activated during myelopoiesis could contribute to lipid accumulation in MDSCs thereby enhancing their immunosuppressive function. Despite the progress made in studying this signaling pathway, how mTOR affects the immunosuppressive function of MDSCs is yet to be substantiated.

### STAT

Members of the signal transducer and activator of transcription (STAT) protein family has been reported to regulate MDSCs functions by coordinating various activities (112). STAT3 signaling is crucial for the activation and expansion of MDSCs in several pathophysiological conditions (113) (Figure 3). STAT3 and STAT5 signaling induced by G-CSF and GM-CSF, respectively, regulates the expression of proteins critical for expansion, differentiation and activation of MDSC (32). Recently, Al-Khami et al., demonstrated that the pharmacological blockage of STAT3 by FLLL32 or STAT5 by pimozide in BM-derived MDSCs decreased the level of intracellular accumulated neutral lipids. More importantly, these inhibitors prevented the induction of arginase-1 and iNOS, thereby abrogating the development of immunosuppressive functions of MDSCs. The effects of STAT3 and STAT5 inhibition suggest the role of lipids in driving the immunosuppressive function of MDSCs (25). Despite the available evidence, there is still a gap in understanding how STAT3/STAT5 signaling regulates lipid metabolism and immunosuppressive mechanisms in MDSCs.

Our group previously discovered that culturing mouse bone marrow-derived MDSCs in the presence of LA, elevated MDSCs proliferation while co-treatment with JS1-124 (STAT3 inhibitor) reversed this effect. MDSCs generated *in vitro* with or without JSI-124 or LA treatment was co-cultured with allogenic T-cells for 3 days to evaluate the influence of STAT3 on MDSCs suppressive activity by CFSE dilution. The proliferation of T-cell was elevated in the co-treated group compared to LA treated group (55). A marked decrease was observed in the suppressive ability of MDSCs generated in the presence of LA and JS1-124-treated cells (55). Furthermore, STAT3 inhibition or blockage in the expression of STAT3 in conditional knockout mice led to a decline in MDSCs number and improved T-cell response in tumor (114).

The transcriptional factor STAT5 can be activated by GM-CSF which has a key role in myelopoiesis and expansion of MDSCs (115). It was recently reported that GM-CSF in PMNs control the expression of FATP2 through the phosphorylation of STAT5 (26). Deletion of STAT5 in PMNs slowed tumor growth in comparison with the control mice. This was associated with a decreased expression of FATP2 in PMNs. Hence, STAT family could be another potential target for the immunosuppressive activity of MDSCs via fatty acid regulation.

### TIPE Family

Tumor necrosis factor alpha-induced protein 8 like (TIPE or TNFAIP8L) family is a group of recently established regulators of tumorigenesis and immunity (116, 117). There are four homologous mammalian members of this family that have been identified including TIPE (the primary member of the family), TIPE1, TIPE2, and TIPE3 (118). TIPE and TIPE1 are ubiquitously expressed members, TIPE2 is found in the hematopoietic cells and TIPE3 expression is restricted to secretory epithelial tissues.

TIPE family was reported as the only defined transfer protein of second messenger molecules, phosphatidylinositol 4,5-bisphosphate (PIP2) and PIP3 (119, 120). Research revealed the involvement of TIPE family in the transport of phospholipids in and out of the plasma membrane via signaling of phosphoinositide to regulate PI3K (119) (Figure 3). Activation of PI3K can also stimulate the STAT3 pathway which is related to cancer and inflammation (121–123) as well as the NF-κB signaling (124, 125). These signaling pathways play important roles in lipid metabolism and contribute to the immunosuppressive activities of MDSCs (25, 55, 100, 101).

TIPE2 expression is most abundant in the hematopoietic cells (including MDSCs) and T-cells. Upon lipopolysaccharide (LPS) stimulation, nitric oxide production was increased due to loss of TIPE2 gene in macrophages (126). Likewise, it modulates macrophage response to oxidized low-density lipoproteins (ox-LDL). Its deficiency in macrophage enhanced the stimulation of inflammatory cytokines which induced oxidative stress associated with p38, NF-κB, and JNK signaling (127). In relation to these findings, TIPE2 deficient bone marrow increased the formation of atherosclerosis in Ldlr^−/−^ mice consuming ox-LDL and high-fat diet, suppressed TIPE2 mRNA expression (127). Exploring the crosstalk of lipid metabolism in MDSCs with TIPE2 could be another promising approach in cancer therapy.

### PGE2

PGE2 is a pro-inflammatory molecule elicited by stromal, cancer, infiltrating myeloid cells, and associated with G-protein-coupling receptors (GPCRs). PGE2 is an important bioactive lipid and active product of cyclooxygenase 2 (COX-2). COX-2 catalyzes metabolic pathway involving the transformation of AA to an unstable intermediate PGG2, then to endoperoxide H2 (PGH2) and later into five primary prostanoids (TXA2, PGD2, PGE2, PGF2α, and PGI2) through cell-specific synthase (128). In MDSCs, PGE2 signals via the PGE2 receptor, E-prostanoid 4 (EP4) and upregulates arginase 1 activity in this cell, thereby enhancing its immunosuppressive role (129, 130).

Production of COX-2 increased MDSCs proliferation (Figure 3) correlated to an upregulation in the expression of arginase-1 and iNOS in murine tumor-infiltrating leukocytes (131), thereby promoting tumor. Excess COX-2 stimulated the proliferation of malignant cells, thus compromised tumor immunity (132). PGE2/COX-2 signaling was involved in the differentiation of DC into MDSCs in an *in vitro* study (133). This impaired DC maturation and its antigen presentation ability, thus inhibiting MHC class II expression and T cell activation (134). Exploring COX-2 expression as a potential target could be a means to enhance immune surveillance and cancer therapy.

Studies on renal carcinoma cells (RCC) revealed the immunosuppressive influence of PGE2 on tumor cells through induction of arginase activity in MDSCs (135). This hindered T-cell activity in the tumor microenvironment resulting from the unavailability of L-arginine (136). Therefore, manipulation of PGE2 expression in MDSCs could enhance immunotherapy (137).

CXCR4 expression in differentiating MDSCs was elevated by the involvement of PGE2 in mice tumor cells (138, 139). Obermajer et al., established in human ovarian cancer that tumor-related PGE2, induced CXCL12 chemokine production and the expression of CXCR4 on MDSCs. The study also reported that PGE2 promotes COX2 expression in MDSCs. However, exposure of MDSCs from ovarian cancer cells to COX2 inhibitors decreased CXCR4 expression and sensitivity to recombinant CXCL12 (140). PGE2 possibly drives MDSCs accumulation by mobilizing chemokines to attract them from the circulation into the tumor microenvironment. COX-2 selective inhibitors and conventional non-steroidal anti-inflammatory drugs (NSAIDS) have previously been established to suppress immune evasion in tumors. It has been proposed that COX-2 inhibitors may stimulate type 1 immune responses by inhibiting MDSCs function (141).

A recent study reported that FATP2 promoted the suppressive role on PMN-MDSCs through the synthesis of PGE2, following exogenous uptake of AA (26). This suggests that regulation of the metabolic pathway involved in the transformation of AA to PGE2 via COX-2 may be a promising path in controlling lipid accumulation and attenuate the immunosuppressive function of MDSCs.

## Conclusions

Metabolic alteration in cancerous cells has long been reported, however, a salient question yet to be fully investigated is the metabolic fate of tumor-associated immune cells. A better understanding of immunosuppression from a metabolic perspective may enhance the identification of new immunotherapeutic targets (142). Lipid metabolic reprogramming of MDSCs is a major contributing factor to its altered phenotype and co-opted immunosuppressive function. In MDSCs, the factors regulating the shift from glycolysis to FAO-OXPHOS in the tumor milieu and the molecular or transcriptional networks controlling its immunosuppressive role have not been fully explored. Understanding the precise roles of different forms of lipids in the tumor microenvironment is challenging. More research focus on elucidating lipid metabolism in MDSCs may enhance the development of therapies to treat cancer in the clinic.

## Author Contributions

DY, AA, and XW developed the study and wrote the paper. MX, LA, GZ, and YC contributed to the critical suggestion.

### Conflict of Interest Statement

The authors declare that the research was conducted in the absence of any commercial or financial relationships that could be construed as a potential conflict of interest.
